# Dynamic radiomics for predicting the efficacy of antiangiogenic therapy in colorectal liver metastases

**DOI:** 10.3389/fonc.2023.992096

**Published:** 2023-02-06

**Authors:** Hui Qu, Huan Zhai, Shuairan Zhang, Wenjuan Chen, Hongshan Zhong, Xiaoyu Cui

**Affiliations:** ^1^ College of Medicine and Biological Information Engineering, Northeastern University, Shenyang, Liaoning, P.R, China; ^2^ Department of Interventional Radiology, First Affiliated Hospital of China Medical University, Shenyang, Liaoning, China; ^3^ Key Laboratory of Diagnostic Imaging and Interventional Radiology of Liaoning Province, First Affiliated Hospital of China Medical University, Shenyang, Liaoning, China; ^4^ Department of Gastroenterology, First Affiliated Hospital of China Medical University, Shenyang, China; ^5^ Department of Medical Oncology, The First Affiliated Hospital of China Medical University, Shenyang, Liaoning, China; ^6^ Key Laboratory of Intelligent Computing in Medical Image, Ministry of Education, Shenyang, China

**Keywords:** colorectal cancer liver metastases, radiomics, dynamic radiomics, antiangiogenic therapy, efficacy prediction

## Abstract

**Background and objective:**

For patients with advanced colorectal liver metastases (CRLMs) receiving first-line anti-angiogenic therapy, an accurate, rapid and noninvasive indicator is urgently needed to predict its efficacy. In previous studies, dynamic radiomics predicted more accurately than conventional radiomics. Therefore, it is necessary to establish a dynamic radiomics efficacy prediction model for antiangiogenic therapy to provide more accurate guidance for clinical diagnosis and treatment decisions.

**Methods:**

In this study, we use dynamic radiomics feature extraction method that extracts static features using tomographic images of different sequences of the same patient and then quantifies them into new dynamic features for the prediction of treatmentefficacy. In this retrospective study, we collected 76 patients who were diagnosed with unresectable CRLM between June 2016 and June 2021 in the First Hospital of China Medical University. All patients received standard treatment regimen of bevacizumab combined with chemotherapy in the first-line treatment, and contrast-enhanced abdominal CT (CECT) scans were performed before treatment. Patients with multiple primary lesions as well as missing clinical or imaging information were excluded. Area Under Curve (AUC) and accuracy were used to evaluate model performance. Regions of interest (ROIs) were independently delineated by two radiologists to extract radiomics features. Three machine learning algorithms were used to construct two scores based on the best response and progression-free survival (PFS).

**Results:**

For the task that predict the best response patients will achieve after treatment, by using ROC curve analysis, it can be seen that the relative change rate (RCR) feature performed best among all features and best in linear discriminantanalysis (AUC: 0.945 and accuracy: 0.855). In terms of predicting PFS, the Kaplan–Meier plots suggested that the score constructed using the RCR features could significantly distinguish patients with good response from those with poor response (Two-sided P<0.0001 for survival analysis).

**Conclusions:**

This study demonstrates that the application of dynamic radiomics features can better predict the efficacy of CRLM patients receiving antiangiogenic therapy compared with conventional radiomics features. It allows patients to have a more accurate assessment of the effect of medical treatment before receiving treatment, and this assessment method is noninvasive, rapid, and less expensive. Dynamic radiomics model provides stronger guidance for the selection of treatment options and precision medicine.

## 1 Introduction

Colorectal cancer (CRC) is the fourth most common malignancy worldwide, with approximately 800,000 newly diagnosed cases each year (1). CRC accounts for approximately 10% of all tumors (2). The liver is the most common metastatic site for CRC, and approximately a quarter of all patients with CRC have liver metastases (3, 4). Surgery is the best treatment for colorectal cancer liver metastases (CRLMs). At present, judging whether CRLM patients can undergo surgery is mainly based on two aspects: “technical” and “oncological”. For the “technical” definition of resectable CRLM, the current consensus is that complete macroscopic resection is feasible while maintaining at least 30% of future liver remnants (FLRs) or a residual liver to body weight ratio >0.5. The “oncological” criteria for resectable CRLM mainly consider that patients can achieve higher disease-free survival and cure rate, and based on the number of this lesion ≥ 5, concomitant unresectable extrahepatic lesions and tumor progression are contraindications for surgery in patients with CRLM (5). Under these criteria surgical resection can only be applied to a limited number of cases, and the probability of postoperative recurrence of the liver is extremely high (6). Inhibition of angiogenesis during tumor growth is the standard treatment for unresectable CRLM. Antiangiogenic drugs (e.g., bevacizumab) are currently used in combination with chemotherapy in patients with CRLM (7). However, the patient response to this treatment varies, and there are currently no good indicators for predicting the efficacy of treatment (8). Therefore, it is important to accurately and noninvasively predict the response of CRLM patients to the initial treatment.

Radiomics is a promising and noninvasive method that analyzes traditional medical images to extract quantifiable data, which show the biological characteristics of pathological processes at the microscopic level (9, 10). These data can be converted into image-based signatures to improve the accuracy of diagnosis, prognosis and prediction of cancer patients. Computed tomography (CT) has the advantages of repeatability, standardization, and extraction of quantitative data. It is indispensable in diagnosis and follow-up (11). Although some PET and MRI based radiomics studies have achieved remarkable results in the field of metastatic colorectal cancer (12, 13), CT based imaging criteria are still the preferred criteria for evaluation of tumor drug response in clinical trials so far. CT-based radiomics has been shown to help predict therapy response and outcome in multiple cancers, including CRC (14–16). Ligero et al. verified that their established CT-based radiomics signature is associated with the response of a variety of advanced solid tumors to immune checkpoint inhibitors (17). Jain et al. predicted the overall survival (OS) and response to chemotherapy of small cell lung cancer (SCLC) patients based on the radiomic features within and around lung tumors extracted from CT images (18). In predicting the efficacy and prognosis of CRLM after treatment, Wei et al. constructed a deep learning-based radiomics model using CT images to predict the response of CRLM to advanced first-line chemotherapy, with an AUC of 0.935 in the validation cohort (19); Liu et al. constructed a CT-based radiomics model to predict the survival of unresectable colorectal liver metastases treated with hepatic arterial infusion chemotherapy, and the c-index of the test group reached 0.743 (20).

On the other hand, although various imaging modalities such as ultrasound (US), computed tomography (CT), magnetic resonance imaging (MRI), and positron emission tomography/computed tomography (PET/CT) can be used for the diagnosis and evaluation of CRLM, CT is still the current method of choice for the diagnosis and treatment of CRLM (21, 22). Previous studies have shown that the sensitivity and specificity of CT for the diagnosis of CRLM are 82.1% and 73.5%, respectively (23).

Existing radiomics features were mainly analyzed based on static medical images at one time point. However, the occurrence and development of tumors is a dynamic process, and static image features cannot contain more dynamic information. For this reason, Carvalho et al. proposed “delta radiomics”, which can represent the change in radiomics characteristics over time (24). This approach can provide additional information to identify, quantify, and potentially predict treatment-induced changes during treatment and has been shown to have potential for predicting treatment efficacy and prognosis in colorectal (25) esophageal (26), pancreatic (27), and lung (28) cancers. To improve the workflow and specific techniques of radiomics related to time series, Qu et al. proposed a feature extraction method called dynamic radiomics (29, 30). This method can use multiple series of images from the same type of imaging examination to jointly extract features to delineate the changes in features over time.

For antiangiogenic therapy, the number of blood vessels in the tumor is a common indicator used to evaluate its efficacy (31). In the process of contrast-enhanced CT (CECT), after intravenous injection of contrast medium, tumor vascularity can be effectively observed by comparing the images acquired at different vascular phases (32), while dynamic radiomics features can reflect the changes in the scanned images at different periods and then indirectly evaluate the vascularity of tumors. Therefore, this method is suitable for assessing the efficacy of antiangiogenic therapy. In this retrospective study, dynamic radiomics was applied to predict the efficacy of antiangiogenic therapy for the first time. Compared with conventional radiomics, the model constructed by this method can more accurately predict patient response to treatment and progression-free survival (PFS). Achieve more efficient and precise assessment of patients before they receive treatment. It is helpful for clinicians to make clinical decisions and stratify patients’ prognosis.

## 2 Materials and methods

### 2.1 Patients

The entire cohort was enrolled from June 2016 to June 2021 by reviewing records of the institutional Picture Archiving and Communication System (PACS, Philips) for the identification of patients with histologically confirmed CRLM. A total of 76 patients were confirmed to meet the criteria and all included patients were from single center. The inclusion criteria for this study were as follows: (1) patients were older than 18 years; (2) colorectal adenocarcinoma with liver metastasis was confirmed by histopathological examination; (3) no surgery or other therapy prior to first-line treatment; (4) advanced first-line treatment with bevacizumab combined with a standard chemotherapy regimen (FOLFOX/XELOX/FOLFIRI) was used; (5) first-line treatment evaluation information based on Response Evaluation Criteria in Solid Tumors (RECIST) was available; (6) baseline images of abdominal CECT before first-line treatment were available, which needed to include images in the precontrast phase (PP), arterial phase (AP), portal venous phase (PVP) and delay phase (DP); and (7) the interval between abdominal CT examination and histopathological diagnosis was less than 31 days (range 4–30 days). The exclusion criteria were as follows: (1) the patient had more than one primary tumor site; (2) the CT image quality was poor due to patient respiration or motion artifacts; (3) the patient’s margin was too blurred to delineate; (4) the patient’s clinical data were missing; and (5) the patient’s advanced first-linetreatment had not been completed or the best efficacy had not been reached. Clinical information included age, sex, primary tumor location (left-sided, right-sided and rectum), primary tumor size, and serum carcinoembryonic antigen (CEA) and alpha-fetoprotein (AFP) results at baseline.

This retrospective study was conducted in accordance with the principles of the Declaration of Helsinki and approved by the Ethics Committee of the First Affiliated Hospital of China Medical University, with a waiver of the requirement for informed consent based on its retrospective design.

### 2.2 CT protocol

The contrast administration of abdomen CT scans are patient specific and based on clinical guidelines (33). Sixty-four-slice spiral CT scanners were used to collect the image data of the patients according to a standardized scanning protocol (34). The CT manufacturers used included GE, Phillips, Siemens and Toshiba. The acquisition methods of each CT phase are as follows: Routine plain scan was performed to obtain PP, then 1.2-1.5 mL/kg body weight iohexol was injected intravenously with a high-pressure syringe at a flow rate of 2.5 mL/s, followed by a 20-30 mL saline flush. Patients were imaged in the supine position at full inspiration. AP was obtained 30-35 s after intravenous injection of contrast, PVP was obtained 60-75 s after intravenous injection of contrast, and DP was obtained 100-120 s after intravenous injection of contrast. As shown in **Table 1**, the scanning parameters were as follows: tube voltage 120 kVp (range 100-140 kVp), layer thickness 2 mm, matrix 512 × 512, tube current 333 mA (range 100-752 mA), exposure time 751 ms (range 500-1782 ms), and standardreconstruction algorithm.

**Table 1:** Equipment parameters of this study.

Manufacturers: Toshiba, GE, Phillips and Siemen	
Tube voltage: 120 kVp (range 100−140*kVp*)
Slice thickness: 2.0 mm
Matrix: 512×512
Tube current: 333 mA (range 100–752 mA)
Exposure time: 751 ms (range 500–1782 ms)

All steps were in accordance with the Image Biomarker Standardization Initiative (IBSI) standards. The CT images were stored in DICOM format. Prior to radiographic analysis, each image was examined to ensure that the images collected were suitable for analysis (35).

### 2.3 Lesion segmentation

The CT images were anonymized for all personal and institutional data and labeled with random numbers. For each patient, metastatic liver lesion with the largest cross-sectional area and well-defined margin was selected as target lesion for segmentation, and lesions were segmented separately at different phases. The specific process wasas follows: First, all CT images (PP, AP, PVP and DP) of 76 lesions were contoured slice by slice using a soft tissue window (window width: 350 HU, window level: 40 HU) for selected liver lesions using a semiautomatic fast marching segmentation algorithm. Then, the images were manually modified and segmented using open-source 3D-Slicer software (www.slicer.org) by two radiologists with 10 years of work experience to remove adjacent normal tissues or surrounding bile ducts. In case of contradiction, othersenior radiologists (over 20 years of work experience) would assess the tumor mask again for agreement. CT images in DICOM format were imported into 3D-Slicer software, and regions of interest (ROIs) were subsequently exported into Nearly Raw Raster Data (NRRD) and Medical Reality Markup Language (MRML) formats for storage and further analysis.

### 2.4 Feature extraction

The radiomics features of the ROIs were extracted using the “PyRadiomics” package in the Python environment. The extracted radiomics features could be divided into the following categories: first-order features, shape-based features, texture features and wavelet features. First-order features describe the distribution of the ROI’s endogenous intensities (36). Shape-based features capture the intuitive features of the ROI into two-dimensional and three-dimensional sizes and shapes. These features are independent of the grayscale intensity distribution in the ROI. Texture features were extracted based on five texture matrices: (1) gray level co-occurrence matrix (GLCM), (2) gray level size zone matrix (GLSZM), (3) gray level running length matrix (GLRLM), (4) neighboring gray level difference matrix (NGTDM) and (5) gray level dependence matrix (GLDM) (37). Wavelet features refer to the characteristics of different frequency bands extracted from the wavelet decomposition of the image (38). Based on the suggestions of Pyradiomics developers, we used the following initial settings for feature extraction: ‘binWidth’ = 25; ‘Interpolator’ = sitk.sitkBSpline; ‘resampledPixelSpacing’ = [1, 1, 1]; ‘voxelArrayShift’ = 1000; ‘normalize’= True; ‘normalizeScale’ = 100.

### 2.5 Dynamic feature construction

Dynamic radiomics features use the static feature changes of different series of the same imaging examination or different imaging examinations to construct new features that can describe the change rule, which can be expressed as:


(1) $$\phi \left(\psi \left(x\left(t_{1}\right)\right),\psi \left(x\left(t_{2}\right)\right),\cdots ,\psi \left(x\left(t_{k}\right)\right)\right)$$

where *ϕ*(·) represents the conversion from static radiomic features to dynamic radiomic features, *ψ*(·) represents the process of extracting static features from images, and *x*(*t*_*k*_) represents a series of medical images.

According to the number of series collected and the feature extraction method, 5 construction methods of dynamic features are proposed:

(1) Standard discrete (SD) feature:


(2) $$SD\left(\psi \left(x\left(t\right)\right)\right)=\frac{1}{k}\sum_{i=1}^{k}\left|\psi \left(x\left(t_{i}\right)\right)-\psi \left(x\left(\bar{t}\right)\right)\right|$$

(2) Discrete change (DC) feature:


(3) $$DC\left(\psi \left(x\left(t\right)\right)\right)=\left(\frac{1}{k}\sum_{i=1}^{k}\left|\psi \left(x\left(t_{i}\right)\right)-\psi \left(x\left(\bar{t}\right)\right)\right|\right)/\psi \left(x\left(\bar{t}\right)\right)$$

(3) Relative change rate (RCR):


(4) $$RCR\left(\psi \left(x\left(t\right)\right)\right)=\frac{\left|\psi \left(x\left(t_{j}\right)\right)-\psi \left(x\left(t_{i}\right)\right)\right|}{\psi \left(x\left(t_{i}\right)\right)},1\leq j\leq i\leq k$$

(4) Relative average change rate (RACR):


(5) $$RACR\left(\psi \left(x\left(t\right)\right)\right)=\frac{\left|\psi \left(x\left(t_{j}\right)\right)-\psi \left(x\left(t_{i}\right)\right)\right|}{\psi \left(x\left(\bar{t}\right)\right)},1\leq j\leq i\leq k$$

(5) Ploy (P) feature:


(6) $$\{\begin{array}{c} \hat{\theta }=\arg\min\sum_{i=1}^{k}\left(\psi (x(t_{i}))-m(t_{i},\theta )\right)\\ m(t_{i},\theta )=\sum_{i=1}^{k}a_{i}\cdot t_{i},\theta ={\left(a_{1},a_{2}\cdots a_{7}\right)}^{T} \end{array}$$

where the set *θ* of P features is calculated based on the least-squares estimation model.

### 2.6 Evaluation

The patients were divided into two groups according to the best response to first-line treatment: those who achieved objective response (OR) and those who did not achieve objective response (NOR). Objective response was defined as achievement of complete response (CR) or partial response (PR) according to Response Evaluation Criteria in Solid Tumors (RECIST) criteria version 1.1 (39). Due to the small number of samples included, we employed leave-one-out cross-validation to measure the prediction performance of different features in different algorithms. We used the t test to screen the features that differed between the OR and NOR groups and then used the least absolute shrinkage and selection operator (Lasso) to reduce the dimensionality of the training set to obtain the required features for the training model. For comparison with traditional radiomics, in addition to the five constructed dynamic features, we included the static features of different series and the collection of static features for modeling. In the training cohort, three machine learning methods were used to construct the scores for the prediction of the efficacy of chemotherapy + bevacizumab, including support vector machine (SVM), linear discriminant analysis (LDA) and random forest (RF). Among all kinds of features, the one with the best predictive performance was selected.

The features with the best performance in the classification task were used for univariate Cox regression analysis to select the features related to Progression-free survival (PFS) (P <0.05), and a PFS-based efficacy prediction score was constructed using a random survival forest model. PFS was defined as the time from randomization to the first occurrence of disease progression or death from any cause.

### 2.7 Statistical analysis

The area under the receiver operating characteristic (ROC) curve (AUC) in the validation dataset was analyzed using the “pROC” package in R, and the performance of different prediction scores was compared using the AUC and accuracy. Time-dependent ROC curves were plotted using the ßurvivalROC” package in R, and the predictive performance of the model at 90, 180, 270 and 360 days was evaluated using AUCs. We used the “rms” package to draw nomograms, and calibration curves were used to assess the discriminability of the nomograms. Kaplan–Meier plots were constructed to analyze potential differences in PFS between the high-risk and low-risk groups. All statistical analyses were performed using R (version 4.1.1). Fisher’s exact test was used to determine whether there were significant differences in clinical variables between the OR and NOR groups. Two-sided p values<0.05 were considered statistically significant.

## 3 Results

### 3.1 Patient characteristics

**Figure 1:**
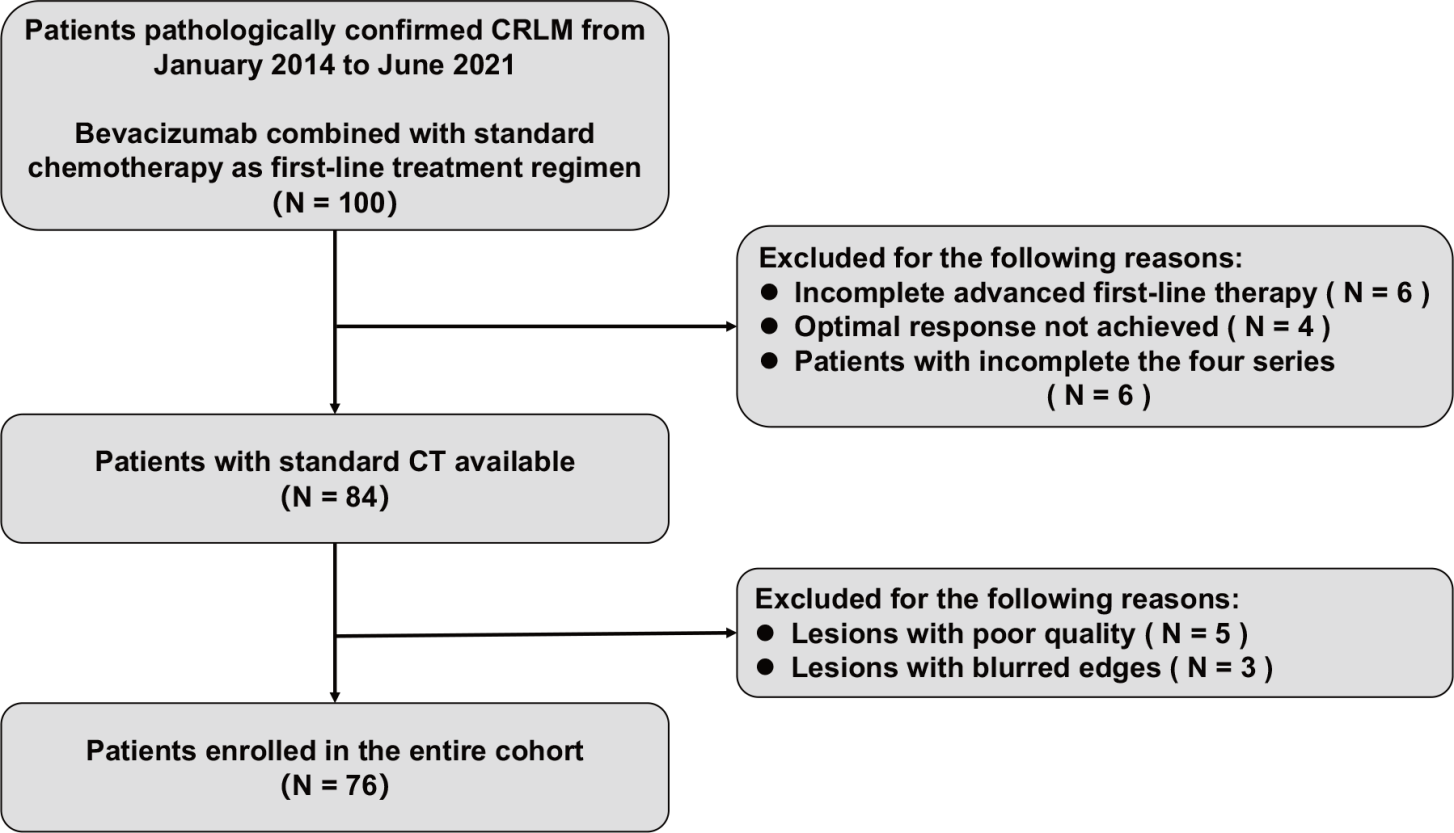
Flow chart of the enrolled patients in the study.

**Figure 2:**
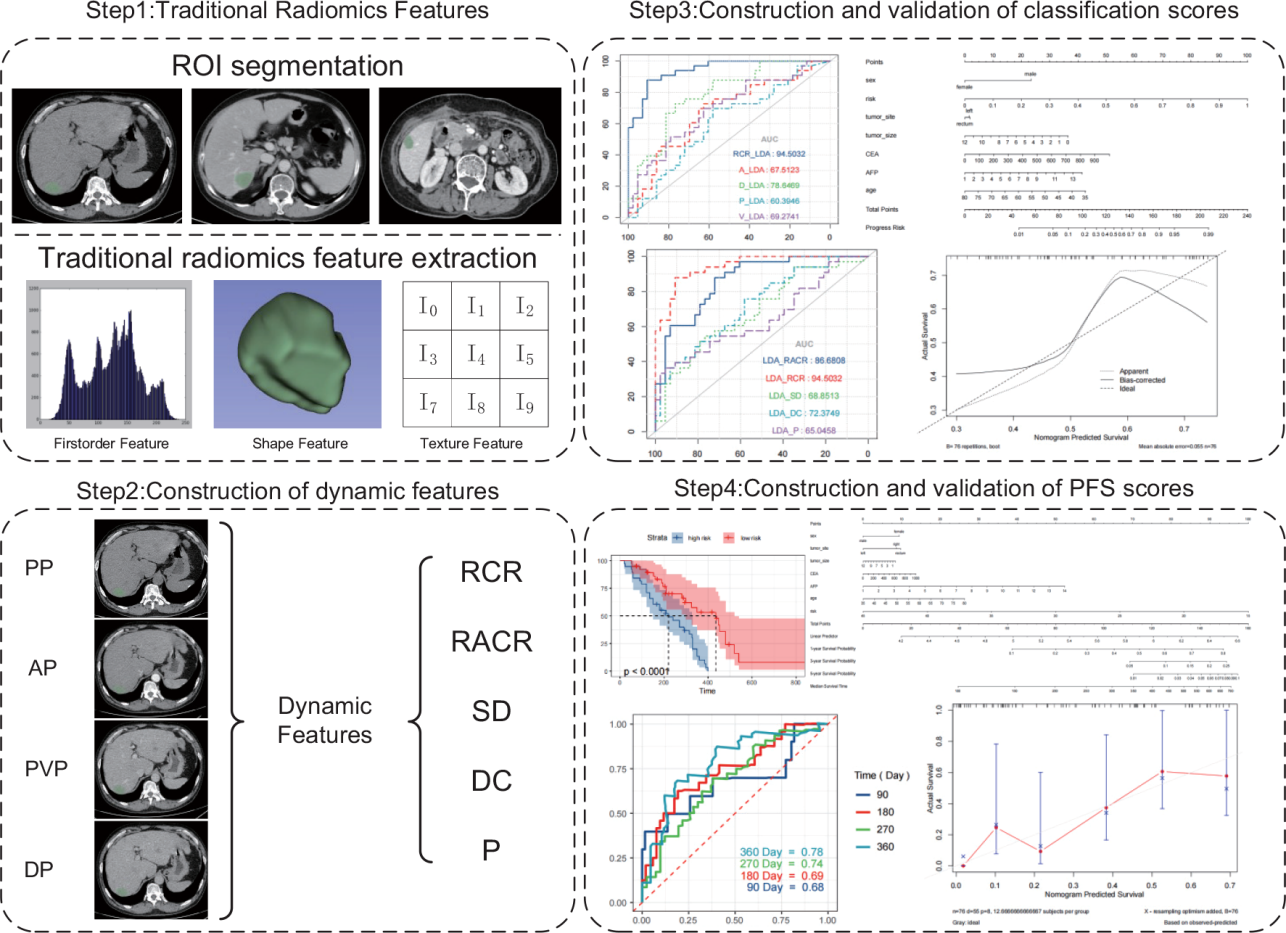
Workflow of the necessary steps in this study.

A total of 76 patients (40 males and 36 females, median age of 60 years, age range between 36 and 76 years) diagnosed with CRLM at the First Affiliated Hospital of China Medical University were enrolled in this study. **Figure 1** shows the patient recruitment process. Based on the best response, the patients were divided into an OR group (33 patients) and an NOR group (43 patients). As shown in **Table 2**, no significant differences in other clinical variables were found between these two groups. Our work flow diagram is shown in **Figure 2**.

**Table 2:** Baseline clinical characteristics of the patients.

	NOR,n(%)	OR,n(%)	P value
Total	43	33	
Sex			
male	20 (46.5)	20 (60.6)	0.323
female	23 (53.5)	13 (39.4)	
Tumor site			
left	11 (25.6)	12 (36.4)	0.597
rectum	17 (39.5)	11 (33.3)	
right	15 (34.9)	10 (30.3)	
Tumor size (mean (SD))	4.28 (2.55)	3.50 (2.15)	0.165
CEA			
normal	4 (9.3)	5 (15.2)	0.671
high	39 (90.7)	(84.8)	
AFP			
normal	41 (95.3)	31 (93.9)	1
high	2 (4.7)	2 (6.1)	
Age			
≤55	11 (25.6)	11 (33.3)	0.629
>55	32 (74.4)	22 (66.7)	

P values were derived from Fisher’s exact test.

### 3.2 Construction and validation of classification prediction scores

After excluding features with the same values in all patients (40), we obtained 1329 radiomic features and constructed dynamic features using static features from four different vascular phases. After performing a t test (P s 0.05), we further screened features on the training set using Lasso. **Table 3** and **Table 4** show the performance of different features in test samples after cross-validation based on the leave-one-out method.

**Table 3:** Prediction AUCs based on various dimensions of LDA, RF and SVM.

	LDA	RF	SVM
RACR	0.867	0.780	0.822
RCR	0.945	0.841	0.908
SD	0.689	0.623	0.662
DC	0.724	0.615	0.716
P	0.651	0.631	0.671
AP	0.675	0.574	0.618
DP	0.787	0.666	0.749
PP	0.604	0.635	0.548
PVP	0.693	0.768	0.591
Multi_static	0.853	0.642	0.817

Multi_static refers to the feature set analysis of multiple series.

**Table 4:** Prediction accuracies based on various dimensions of LDA, RF and SVM.

	LDA	RF	SVM
RACR	0.737	0.684	0.737
RCR	0.855	0.803	0.803
SD	0.658	0.605	0.632
DC	0.645	0.592	0.618
P	0.605	0.645	0.618
AP	0.605	0.540	0.553
DP	0.737	0.658	0.645
PP	0.592	0.526	0.526
PVP	0.618	0.763	0.526
Multi_static	0.763	0.592	0.724

Where Multi_static refers to the feature set analysis of multiple series.

**Figure 3:**
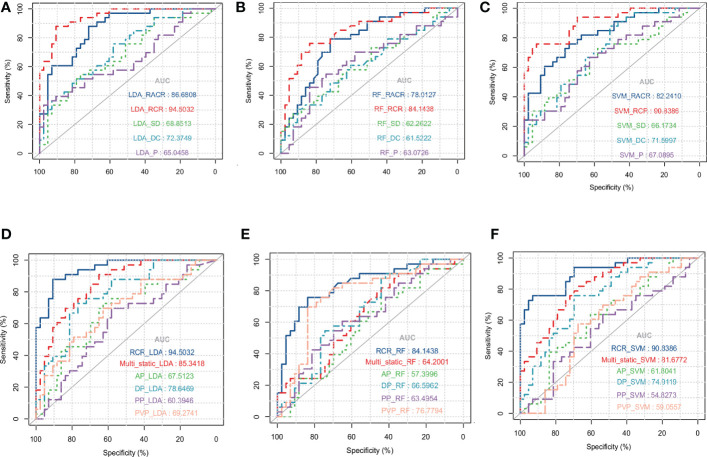
ROC curves for LDA **(A)**, RF **(B)** and SVM **(C)** models with different dynamic features when using leave-one-out cross-validation. ROC curves for LDA **(D)**, RF **(E)**, and SVM **(F)** models with static features of different series and RCR features when using leave-one-out cross-validation, where Multi_static refers to the feature set analysis of multiple series.

Of the three machine learning methods, all five dynamic features showed their best predictive performance in LDA (**Figures 3A–C**). Compared with other dynamic features, RCR features showed the best classification performance in all three machinelearning methods. As shown in **Table 5**, after lasso processing, the RCR features constructed from each of the 16 radiomics features were selected for constructing machine learning models. In the LDA model, the RCR AUC and accuracy in the validation data reached 0.945 and 0.855, respectively. It also had the best performance compared to all static features (**Figures 3D-F**).

**Table 5:** Radiomics features used to construct RCR features obtained after lasso selecting.

Radiomics feature	
wavelet.HHH_glszm_LowGrayLevelZoneEmphasis
wavelet.HHL_glrlm_RunEntropy
wavelet.HLH_glcm_Imc2
wavelet.LHH_firstorder_Median
wavelet.HLL_glcm_MCC
original_shape_Elongation
wavelet.HHH_glszm_SizeZoneNonUniformityNormalized
wavelet.LHH_firstorder_Skewness
wavelet.LLL_glcm_Idn
wavelet.LLH_glszm_SizeZoneNonUniformityNormalized
exponential_glrlm_RunVariance
squareroot_glcm_DifferenceEntropy
wavelet.HLH_glszm_HighGrayLevelZoneEmphasis
wavelet.HLH_ngtdm_Contrast
wavelet.HLL_glszm_LowGrayLevelZoneEmphasis
wavelet.LHL_firstorder_Mean

**Figure 4:**
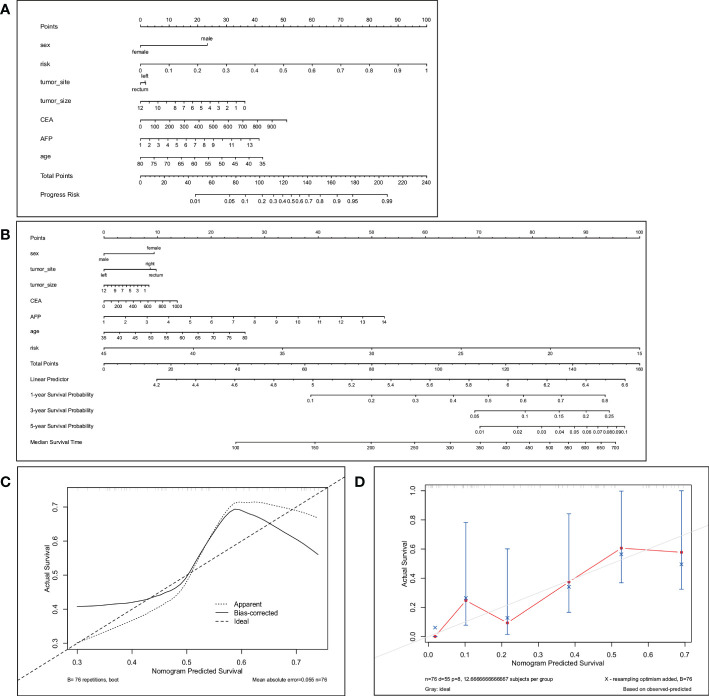
The nomogram **(A)** predicts the best response in patients with CRLM. The total score is calculated by summing the points for each factor. The total score corresponds to the patient’s best response prediction. **(C)** is the calibration curve corresponding to the nomogram. The nomogram **(B)** predicts 1-year, 3-year and 5-year PFS in patients with CRLM. Total points are calculated by summing the points for each factor. The total score corresponds to the 1-, 3-, and 5-year PFS probabilities of the patients. **(D)** Calibration plots to predict 1-year progression-free survival (PFS).

Previous studies have shown that age, sex, and CEA and AFP levels are also factors predicting the efficacy of bevacizumab (41, 42), so we used these variables and our best predictive score (the result of RCR features in the LDA model) to construct a nomogram (**Figure 4A**). The calibration curves of the nomogram showed good agreement between the classification results predicted by the nomogram and the actual observations (**Figure 4C**).

### 3.3 Efficacy prediction model based on PFS

**Figure 5:**
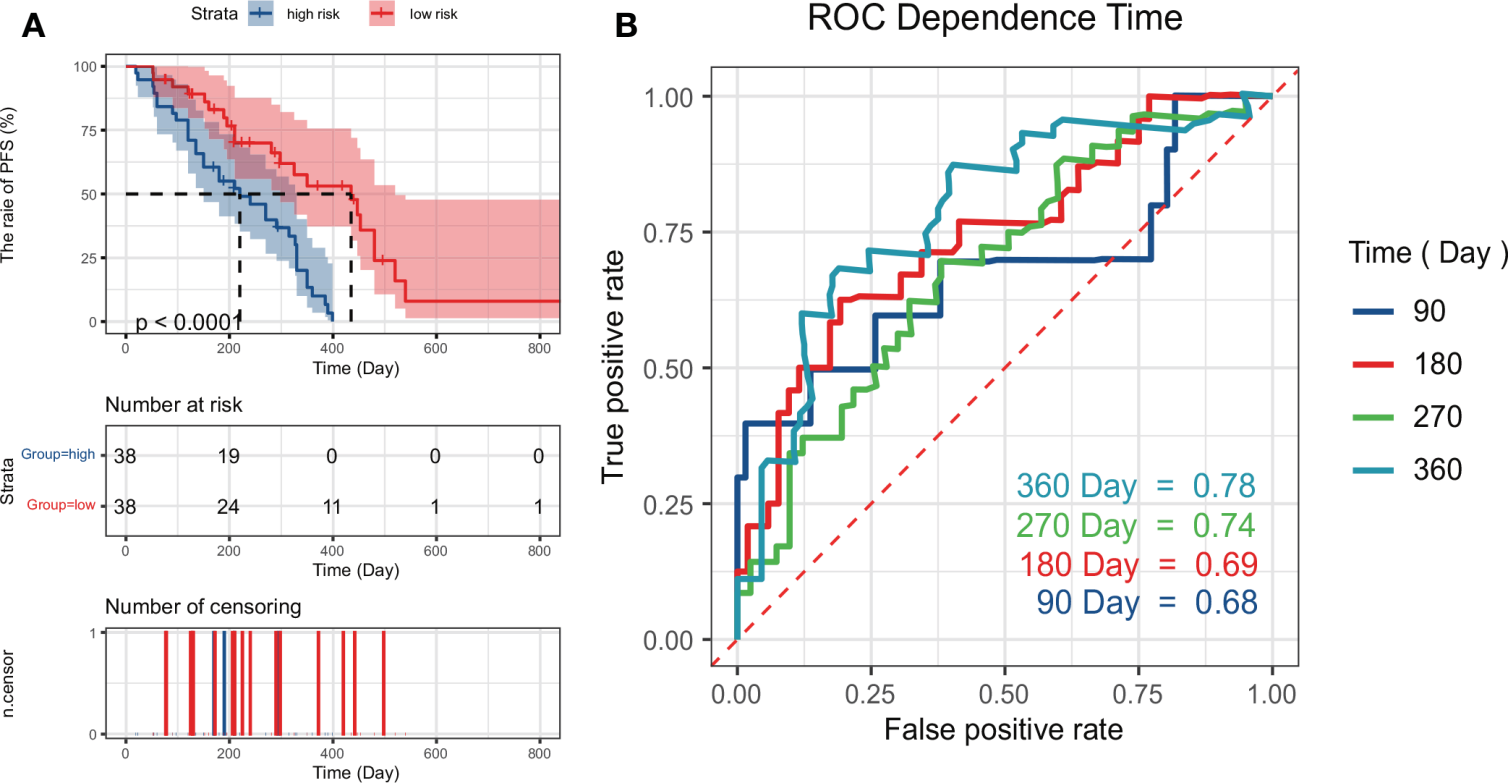
Kaplan–Meier plots **(A)** obtained by dividing patients into high-risk and low-risk groups using the median predictive score of the random survival forest model; **(B)** shows ROC curves estimated at 90, 180, 270, and 360 days using the predictive scores.

We selected the RCR features with the best performance in the classification task, constructed a PFS-based efficacy prediction model using leave-one-out cross-validation + random survival forest, and divided the patients into high- and low-risk groups according to the median risk score. Kaplan–Meier plots demonstrated a significant difference (P <0.0001) in PFS between the two groups (**Figure 5A**). The time-dependent ROC curve indicated that the PFS-based prediction score had good predictive power at different time points (**Figure 5B**). We also constructed a nomogram (**Figure 4B**), and survival calibration plots showed that the survival probabilities predicted by the nomogram also had good agreement with the actual observations (**Figure 4D**).

## 4 Discussion

In this study, we use a new dynamic radiomics feature extraction method and workflow based on multiple series. The extraction of all dynamic features is based on static feature extraction, which describes the variation of static features at different times. In the study by Qu et al., it had been confirmed that dynamic radiomics had better predictive performance compared with traditional radiomics in the tasks of tumor diagnosis prediction, tumor patient gene mutation status prediction and patient prognosis prediction (29). We used this method to predict both the response to antiangiogenic therapy and PFS in patients with CRLM. Compared with traditional radiomics, the prediction performance of dynamic features is greatly improved and superior to that of clinical predictors (36, 37) (**Figure S1**, **Figure S2**).

In the field of CRC, radiomics has been widely used for diagnosis and predicting prognosis and the efficacy of drugs (43–45). In recent years, the analysis of CRLM using image features extracted by deep learning has also been common in radiology. Shi et al. used an artificial neural network (ANN) model to predict the mutation status of RAS and BRAF genes in CRLM patients (35). Zhu et al. used deep learning-assisted magnetic resonance imaging to predict tumor response to chemotherapy in CRLM patients (46). Starmans et al. used deep learning to differentiate the pure histopathological growth patterns of CRLM on CT (47). Compared with deep learning and traditional radiomic features, dynamic features have the following advantages. (1) Compared with traditional radiomic features, dynamic features can reflect changes in the static features of all sequences, so this method can extract more features and information for model construction. (2) Dynamic features calculate the relative changes in static features. Therefore, dynamic features are less affected by image quality differences between different series of the same patient or between different patients. (3) Compared with deep learning features, dynamic features are easier to interpret and therefore more acceptable to doctors (48). (4) Compared with deep learning, it is suitable for small sample research and more suitable for medical (49). (5) Different sequences of medical images are considered, which is more consistent with the actual image diagnostic process of doctors (21).

In this study, the RCR feature had the best prediction performance among all models, which may be because it contains the relationship between any two sequences, contains the most features and has less information about the whole population. Further research is needed on how to optimize other dynamic features and explore how to select features for different tasks.

Lesion segmentation is a critical task for both radiomics and dynamic radiomics. Stefano et al. discussed the impact of manual segmentation and semi-automated segmentation on radiomics studies in their study (50), and the authors concluded that manual flexible delineation of targets allows highly accurate segmentation. However, manual segmentation is labor-intensive and time-consuming and is less feasible due to tasks with large data. Moreover, manual segmentation results are easily influenced by observer subjectivity. Therefore, many semi-automatic delineation algorithms are applied in practice, such as region growing or thresholding. But the result of semi-automatic segmentation is not as precise as manual segmentation. In this paper, we used manual segmentation to delineate ROIs for the following reasons: (1) the data volume in this study was small, which requires us to minimize bias as much as possible in the operation, while the results of manual segmentation are more accurate; (2) the additional workload due to the use of manual segmentation in this study is acceptable; (3) in order to reduce the influence of subjective factors on the segmentation results, each lesion was segmented independently by two radiologists. Once their segmentedresults were quite different, a senior physician would adjudicate the results to ensure the accuracy of the results.

As Pasini et al. reported in their study (51), due to the use of four different CT scanners, some analysis is necessary to assess whether there is a batch effect. For this reason, we performed principal component analysis (PCA) on the RCR features finally adopted in this paper, and the results are shown in **Figure S3**. No significant batch effects was observed among the data collected by different scanners. Therefore, we did not use any statistical harmonization methods such as ComBat to calibrate the data.

Despite the good results achieved by dynamic features, our study still has some limitations. First, the data in this study were collected retrospectively. Secondly, although omitted leave-one-out cross-validation was used to test the performance of models, the insufficient sample size may still lead to the bias of the results, requiring very large datasets and multicenter data for prospective investigation to further verify the robustness and reproducibility of our conclusions. Despite these limitations, we believe that the results obtained in this study are credible and can be extended to a larger patient population.

## 5 Conclusion

In this study, dynamic radiomic feature extraction and workflow were used to predict the efficacy of advanced first-line chemotherapy combined with antiangiogenic therapy in patients with CRLM. While retaining the advantages of traditional radiomics, such as non-invasive, rapid and inexpensive, the dynamic radiomics model achieved higher accuracy than radiomics in predicting both optimal efficacy and PFS. The application of dynamic radiomics to predict the efficacy of antiangiogenic therapy has strong clinical significance and broad development prospect.

## Data availability statement

Due to privacy restrictions, the datasets presented in this article are not publicly available. Requests to access the datasets should be directed to Xiaoyu Cui, cuixy@bmie.neu.edu.cn.

## Ethics statement

The studies involving human participants were reviewed and approved by the First Affiliated Hospital of China Medical University. Written informed consent for participation was not required for this study in accordance with the national legislation and the institutional requirements.

## Author contributions

Conceptualization, XC and HSZ; methodology, HQ; validation, HQ, SZ and HZ; formal analysis, HQ and SZ; resources, HZ; data curation, WC; writing—original draft preparation, HQ; writing—review and editing, HZ; supervision, XC; project administration, HSZ; funding acquisition, HSZ and XC. All authors contributed to reviewing and revising the manuscript.
