# Supplementary material for: Dynamic radiomics for predicting the efficacy of antiangiogenic therapy in colorectal liver metastases
Source: Front Oncol. 2023 Feb 6;13:992096. doi: 10.3389/fonc.2023.992096 (PMC9939899; doi:10.3389/fonc.2023.992096)
Supplement: Supplementary file 1 [file DataSheet_1.pdf]

Supplementary Material

1 SUPPLEMENTARY FIGURES

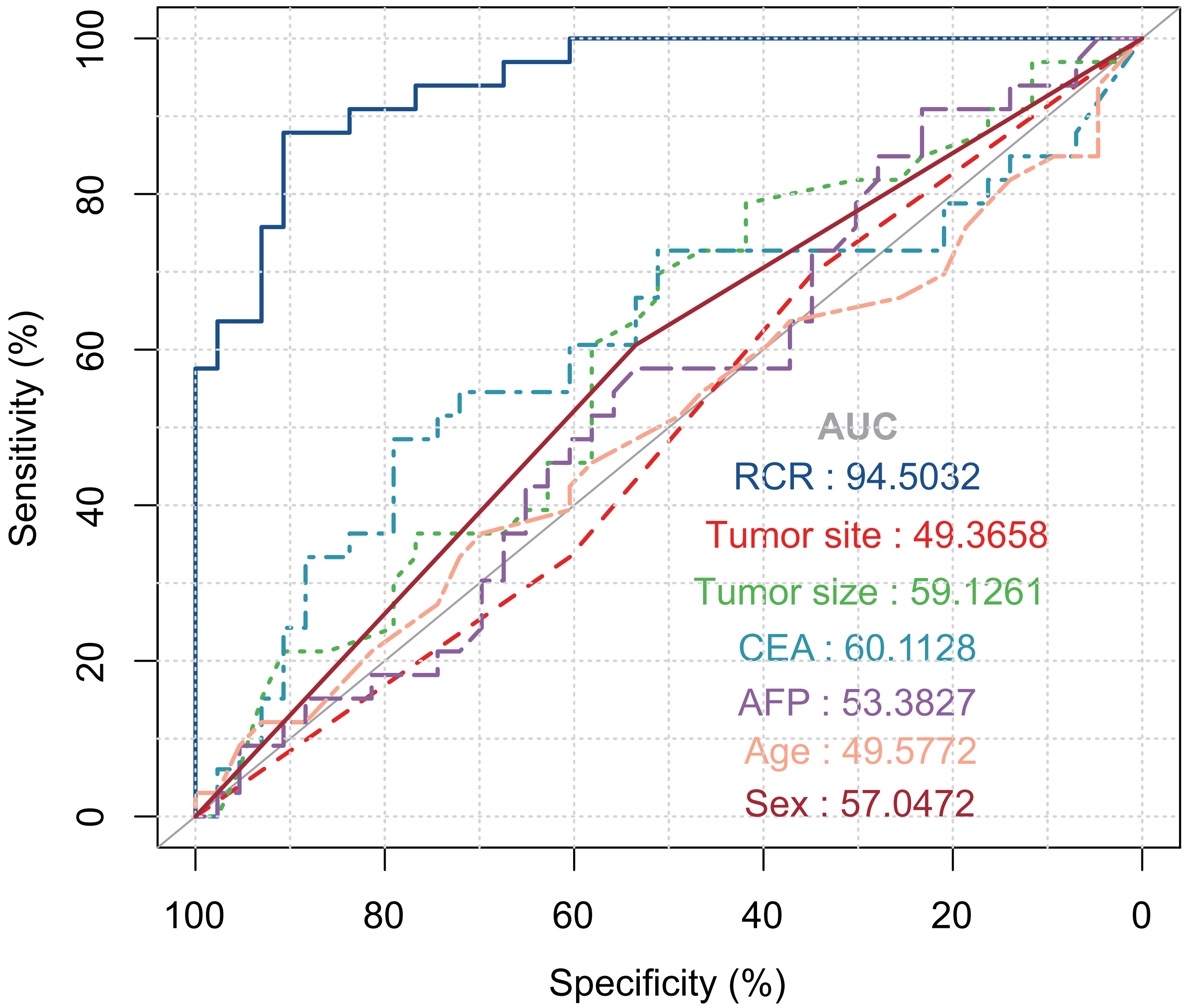

Figure S1. ROC curves for RCR features using scores established by LDA and other clinical predictors.

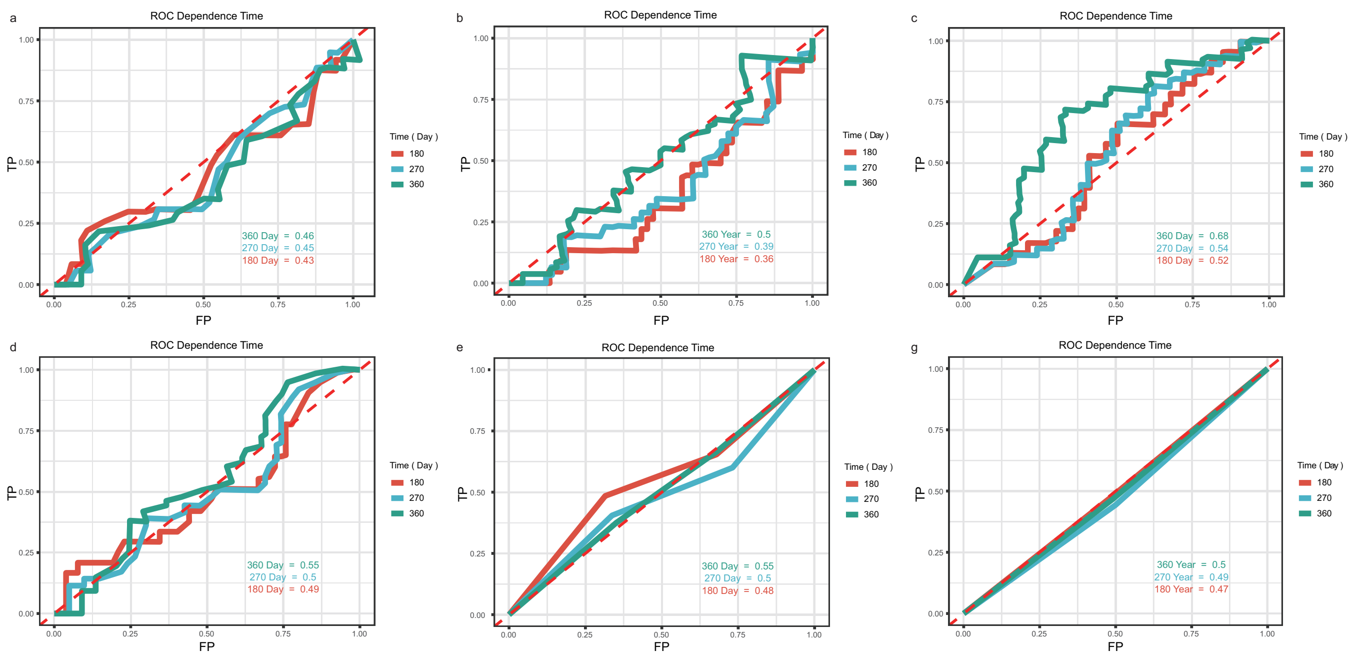

Figure S2. Time-dependent ROC curves for Age (a), AFP (b), CEA (c), Tumor size (d), Tumor site (e) and Sex (g).

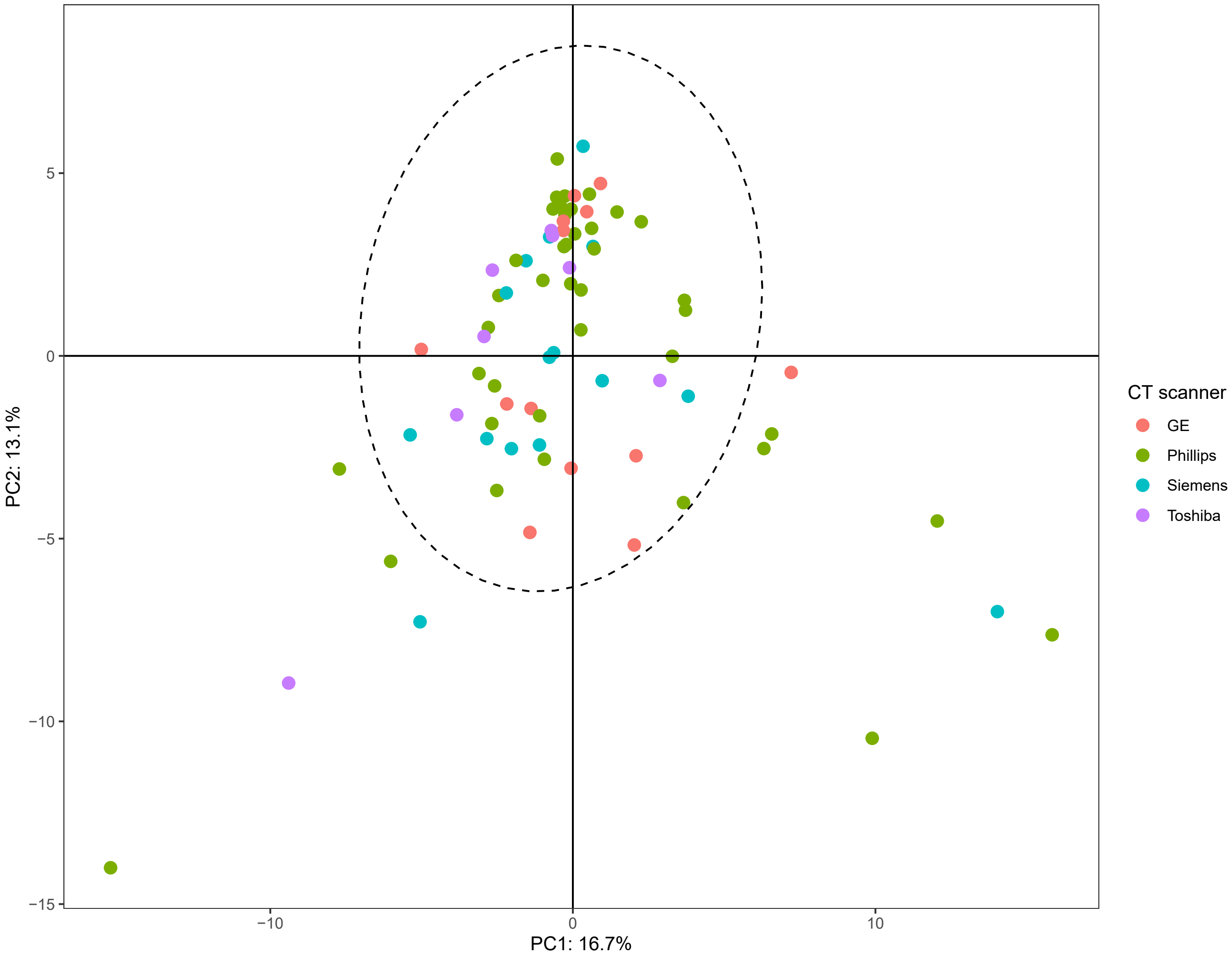

Figure S3. PCA for RCR features.
